# Cell-Free Systems to Mimic and Expand Metabolism

**DOI:** 10.1021/acssynbio.4c00729

**Published:** 2025-01-29

**Authors:** Blake J. Rasor, Tobias J. Erb

**Affiliations:** † Department of Biochemistry and Synthetic Metabolism, Max Planck Institute for Terrestrial Microbiology, 35043 Marburg, Germany; ‡ Center for Synthetic Microbiology (SYNMIKRO), 35043 Marburg, Germany

## Abstract

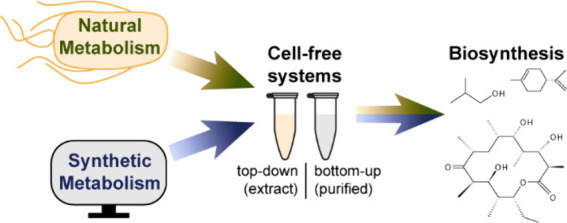

Cell-free synthetic biology incorporates purified components and/or crude cell extracts to carry out metabolic and genetic programs. While protein synthesis has historically been the primary focus, more metabolism researchers are now turning toward cell-free systems either to prototype pathways for cellular implementation or to design new-to-nature reaction networks that incorporate environmentally relevant substrates or new energy sources. The ability to design, build, and test enzyme combinations *in vitro* has accelerated efforts to understand metabolic bottlenecks and engineer high-yielding pathways. However, only a small fraction of metabolic possibilities has been explored in cell-free systems, and extracts from model organisms remain the most common starting points. Expanding the scope of cell-free metabolism to include extracts from new organisms, alternative metabolic pathways, and non-natural chemistries will enhance our ability to understand and engineer bio-based chemical conversions.

## Introduction

**Figure 1:**
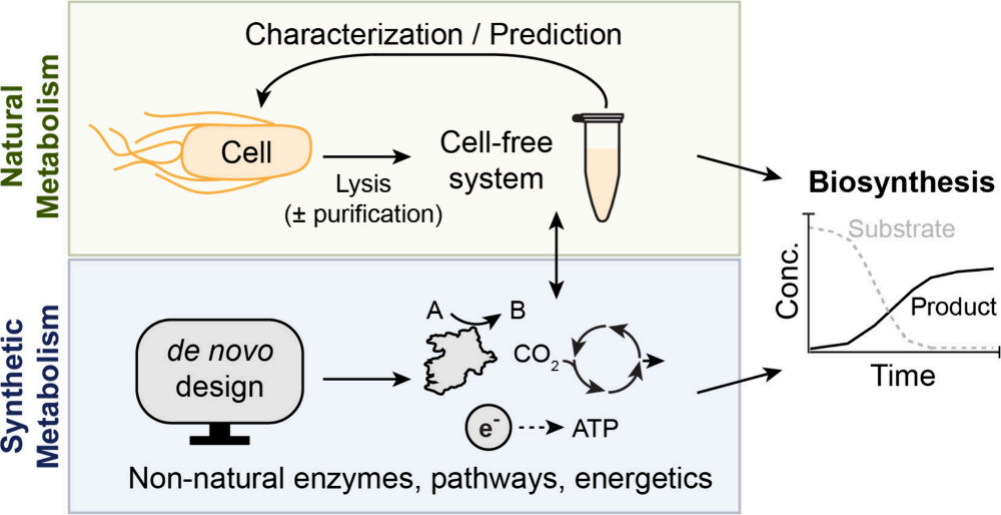
**Overview of the interactions between natural and synthetic metabolism.** Proteins and pathways designed *in silico* can be implemented in cell-free systems for direct applications in metabolic conversions or used to test large sets of constructs prior to optimization *in vivo*.

Cell-free biology enables the rapid study of biochemical pathways in quasi-native metabolic contexts without the confounding impacts of growth or evolution, while offering precise control over these systems that would be impossible to achieve *in vivo*. Cell-free systems can be prepared either bottom-up from purified components or top-down from the soluble extracts of lysed cells.^1,2^ “Purified systems” have well-defined reaction and energy supplying networks, while “cell extracts” represent a snapshot of the metabolic reaction networks of the corresponding cells at the time of lysis, which can rapidly probe biochemical reactions at this particular cell state.^3,4^ Both purified and crude cell-free systems have demonstrated utility for prototyping enzyme variants and enzyme ratios under different conditions.^5−7^ Cell extracts, in particular, have become a powerful and accessible proving ground as synthetic biologists strive to engineer biological systems for predictable and sustainable biochemical conversions.^7,8^ Significant optimization of *Escherichia coli* cell extracts has resulted in different protein and metabolite synthesis schemes that are fueled primarily by glycolysis and produce high yields (∼8 g/L protein, ∼1 M metabolites).^9,10^ At the same time, increasing development of other cell-free biosynthesis platforms, including nonmodel organisms or purified cell-free systems, has enabled the engineering of “non-standard” metabolism^11^ and realization of *de novo* metabolic pathways.^12^ Together, the many types of cell-free systems accelerate synthetic biology with reduced design-build-test cycle times and higher-throughput exploration of biological and chemical diversity.^1,2,13^ This article highlights the utility of cell-free systems in studying and engineering microbial metabolism as well as the ability to move beyond natural biochemistry for sustainable and efficient chemical synthesis *in vitro* (Figure 1).^14^ Selected examples include the accelerated characterization of nonmodel and/or genetically less accessible organisms, the ability to engineer biochemical processes for one-carbon (C1) substrate conversions outside of autotrophs, and the integration of biological systems with sustainable energy sources (e.g., hydrogen, light, electricity, formate dehydrogenase).

## Cell-Free Prototyping Facilitates the Study and Engineering of Metabolism

Recent efforts have exploited the reduced complexity of cell extracts to understand biological functions in a cell-like environment. Now cell extracts facilitate rapid assessment of metabolic pathways and genetic parts in near-native contexts.^15^ In addition to the widely used and commercialized *E. coli* and PURE cell-free systems,^9,16^ cell extracts from dozens of microbes have been adopted for genetic part characterization,^4,17,18^ and some examples extend cell-free metabolism beyond *E. coli* pathways.^11,19^ One noteworthy example is the development of a cell-free gene expression platform from a *Mycoplasma*-derived minimal organism,^20^ which could help to illuminate principles for minimizing the metabolic and genetic components in living systems.^21^ The *in vitro* exploration of additional nonmodel organisms will provide an important avenue for understanding their unique physiology and metabolism for applications in extreme environments, including space exploration.^22,23^ Independent of an extract-based or defined system, one aspect central to the success of cell-free prototyping approaches is to ensure that the cell-free metabolic networks under study closely resemble a relevant *in vivo* state. This makes it important to increase stability of components such as nucleic acids, proteins, and metabolites of interest.^4,15−18^ Therefore, researchers must consider the best context for a given cell-free application and whether to develop new cell-free systems for unique functions or expand the capabilities of existing platforms. For example, post-translational modifications such as glycosylation can occur in eukaryotic extracts with native glycosylation machinery^24^ or be engineered into *E. coli* extracts.^25^ Similarly, extremophilic enzymes may be screened in crude extracts from their native host organism or tested in cell-free systems with modified physical parameters (temperature, salinity, pH).^26,27^

A powerful attribute of well-optimized cell-free systems is that the characterization of genetic parts and metabolic pathways *in vitro* can predict cellular performance *in vivo* with significantly reduced timeframes. Recently, resource competition and growth burden *in vivo* could be predicted from cell extract experiments with high correlations (R^2^ ∼0.75), although this correlation decreased when beta-carotene synthesis was included.^28^ Some metabolic comparisons are even transferrable between species, which is best exemplified by biosynthetic pathway prototyping for *Clostridium autoethanogenum* using cell extracts from *E. coli*. In one study, the rapid design-build-test cycles of cell-free *E. coli* extracts was leveraged to reduce the time and effort to engineer *C. autoethanogenum*, a slow-growing anaerobic bacterium with a limited suite of genetic tools. This included homologue and promoter strength prediction *in vivo* from >200 unique biosynthesis pathways *in vitro*, leading to increased cellular titers of butanol and 3-hydroxybutyrate.^7^ Building on this success, *E. coli* extracts were used in a second study to screen potential competing enzymes for acetone biosynthesis in *C. autoethanogenum*, reducing the set of knockout candidates from 13 targets to 3 in a matter of weeks to accelerate production strain development.^8^

These examples show that metabolic prototyping for fermentation in an anaerobic, autotrophic, Gram-positive organism (*C. autoethanogenum*) worked surprisingly well in an aerobic, heterotrophic, and Gram-negative cell-free *E. coli* system. One hypothesis for this correlation between disparate metabolic contexts is that the focus was set on optimizing an anabolic pathway, which rendered differences in catabolism less significant. In general, cell-free pathway prototyping may yield better results when the extract comes from the same organism, for instance when pathways contain oxygen-sensitive enzymes (e.g., butanol prototyping used aerobic Ter enzymes *in vitro* and anaerobic Bcd-EtfAB complex *in vivo*),^7^ when the catabolic state plays a more prominent role, or when downstream metabolism impacts a target pathway. For example, cell-free prototyping of reverse beta-oxidation enzymes for the synthesis of C4- or C6- acids and alcohols in *E. coli* extract resulted in a significantly higher correlation with *E. coli* fermentations than *C. autoethanogenum* fermentations (r= 0.92 vs 0.46). This may result from the longer pathway having additional metabolic branch points and competitive reactions that differ between the two species. Despite the lower correlations observed in the latter case, the ability to screen over 400 unique enzyme combinations enabled the identification of high-performance enzymes to improve product titers in both species with less *in vivo* engineering.^29^ Thus, the high-throughput capacity of cell-free screening is able to compensate for relatively low correlations.

## Cell-Free Metabolism Currently Utilizes a Small Subset of Natural Pathways

The growing repertoire of cell-free systems has increased our ability to study and characterize aspects of both conventional and nonmodel organisms, overcoming limitations such as slow growth with biochemical exploration *in vitro*. However, the majority of cell-free systems incorporate a limited set of ATP-generating reactions despite the diversity of catabolic pathways present in nature. ATP generation *in vitro* most commonly occurs through substrate-level phosphorylation of glycolyic intermediates (*e,g.*, glucose, phosphoenolpyruvate, 3-phosphoglycerate) or sacrificial substrates (e.g., creatine phosphate), although a recent formulation included ribose and starch as accessory substrates.^9,30^ Extract-based systems may also incorporate oxidative phosphorylation from inverted membrane vesicles that form during cell lysis.^31−33^ ATP generation through oxidative phosphorylation can be driven through the tricarboxylic acid cycle fueled from upstream glycolytic intermediates^30^ as well as downstream metabolites like glutamate^34^ and succinate.^35^ These energy regeneration strategies were also used to power cell-free expression systems from autotrophs despite the presence of the Calvin-Benson-Bassham cycle or the Wood-Ljungdahl pathway.^19,36^ Activating endogenous carbon fixation pathways in cell extracts would enable pathway prototyping for the valorization of C1 substrates with the potential for greater correlations in prototyping campaigns for autotrophic production strains. Molecules including carbon mono- and dioxide, formate, methanol, and methane all present exciting opportunities for *in vitro* transformations using natural and/or synthetic pathways, as discussed below.

Emulating *in vivo* approaches such as heterologous expression and coculture systems with the increased flexibility found *in vitro* could facilitate broader utilization of sustainable C1 substrates by overcoming the slow grow rates observed in engineered strains.^37^ Formate consumption via the reductive glycine pathway^38^ and methanol consumption via the ribulose monophosphate pathway^39,40^ permit *E. coli* to grow with doubling times of ∼8 h. Meanwhile, heterologous expression of the Calvin cycle enables *E. coli* to double in 18 h under 10% CO_2._^41^ Higher growth rates with ambient CO_2_ are possible with the expression of carboxysomes for carbon concentration, but the significant metabolic burden of expressing enzymes and microcompartments results in greater heterogeneity across colonies (12–25 h doubling times).^42^ Generating cell extracts from these strains for cell-free metabolism and gene expression could potentially combine the benefits of C1 metabolism with previously established protocols for *E. coli* cell-free systems for accelerated hypothesis testing. Co-culture systems point to another strategy for combining the metabolic capabilities of different species. Growing cyanobacteria in a single flask with heterotrophs enabled synthesis of proteins or metabolites in various production strains fed by sugar synthesized from CO_2_, and growth competition or inhibition can be limited by tuning culture conditions.^43^ Similarly, an engineered endosymbiont combining the photoautotrophy of *Synechococcus elongatus* with the genetic tools of *S. cerevisiae* was capable of doubling in ∼10 h.^44^ These strategies enable the combination of unique metabolic pathways in a single cell, but biochemical compromises and metabolic burden can hinder growth rates and overall productivity. Such limitations could be avoided *in vitro* by simply mixing cell extracts from disparate organisms, such as cyanobacteria and *E. coli*. While extract mixing has yet to be demonstrated for C1 metabolism, hybrid cell-free systems containing extracts from 2 organisms were successful in gene expression^18^ and in metabolite synthesis.^45,46^ These examples pave the way for more complex *in vitro* combinations of metabolic pathways from multiple organisms.

**Figure 2:**
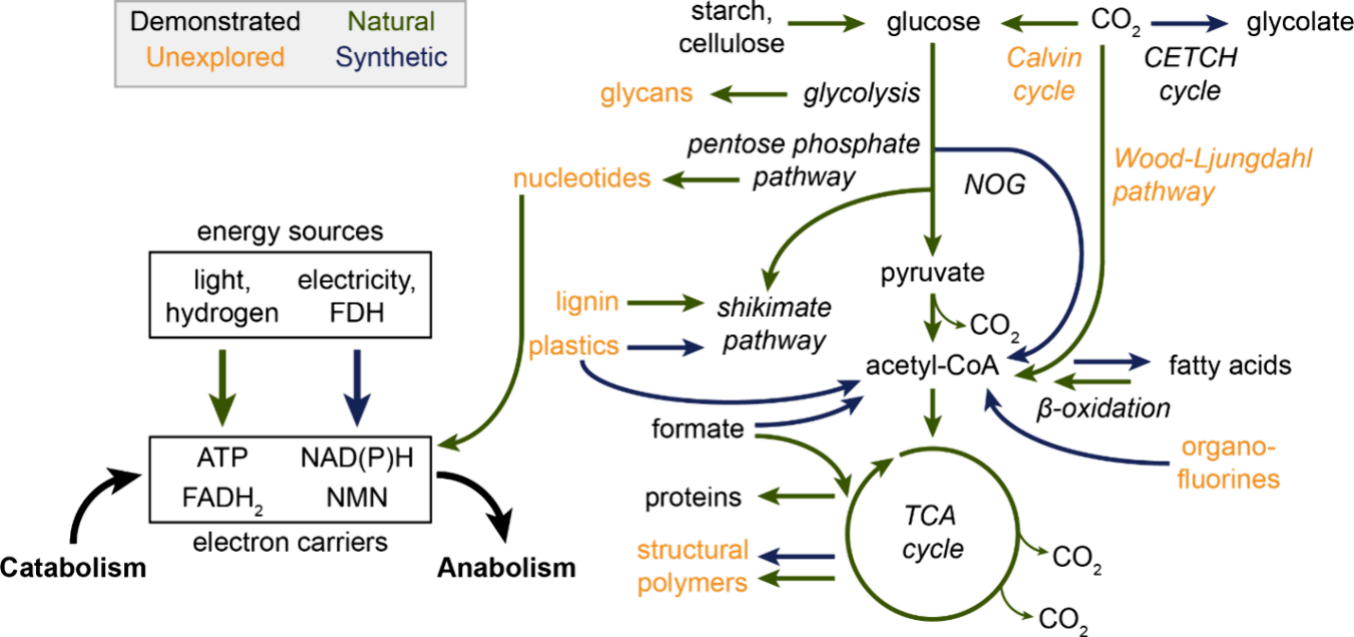
**Key examples of cell-free metabolism in the literature and openings to explore.** Many studies have demonstrated central metabolism *in vitro* with glycolytic intermediates as the predominant substrates. Intermediates such as pyruvate, acetyl-CoA, and glycolate have been converted into diverse products, including terpenes and polyketides. Great potential lies in exploring additional pathways to utilize abundant carbon sources (particularly lignin, C1 gases, and plastics) in combination with sustainable energy sources to further expand the chemical product space. Abbreviations: NOG (nonoxidative glycolysis), FDH (formate dehydrogenase).

Other abundant and sustainable substrates worth exploring with cell-free metabolism include fats/oils,^47^ lignin,^48^ plastic waste,^49^ and organofluorine compounds (PFAS)^50^ for applications in bioremediation and *in situ* resource utilization. The biochemical product space *in vitro* can also be expanded, particularly in the context of cell extracts (Figure 2). Acids, alcohols, and terpenes derived from acetyl-CoA or pyruvate remain the most common products,^2,51^ although recent examples include derivatives of the tricarboxylic acid cycle and amino acid metabolism.^52−54^ Promising outputs for future cell-free chemical conversions include structural biopolymers,^55^ natural or synthetic nucleotides,^56^ and therapeutically relevant glycans.^57^ Incorporating nonmodel organisms, proteins from bioprospecting or metagenomics, and synthetic metabolism approaches will accelerate the diversification of both substrates and products in cell-free metabolism.

## Synthetic Metabolism Enables Novel Biochemical Pathways and Energetics *In Vitro*

**Figure 3:**
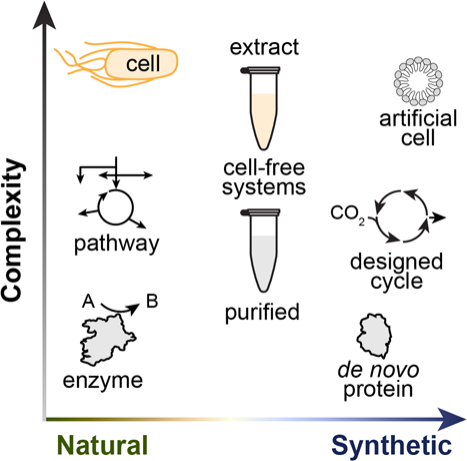
**Landscape of biological parts and systems.** Individual components and multicomponent systems may be fully natural or synthetic. Cell-free systems comprising cell extracts (top-down) or purified components (bottom-up) sit between these ends of the spectrum.

Metabolism research can be imagined on a 2-dimensional spectrum with variable complexity and a range of natural or synthetic origins (Figure 3). In addition to combining natural pathways through heterologous expression or extract mixing, implementing *de novo* designed enzymes and pathways can expand the abilities of biological systems. These designed components or cascades are typically demonstrated in purified cell-free systems but could work together with cell extracts and eventually cellular metabolism. Enzyme design coupled with engineering and selection schemes increasingly enable high-efficiency and new-to-nature chemical reactions, including retro-aldolases tuned from beta-barrel structures^58^ and artificial metalloenzymes catalyzing hetero-Diels–Alder reactions,^59^ hydroamination, or hydroarylation,^60^ which paves the way toward *de novo* C1-converting enzymes in the future. Several new C1-converting enzymes have already been created through the repurposing of active sites.^61−63^ Combinations of these (re)designed enzymes and other enzymes into new C1-converting routes have yielded pathways with increased efficiency compared to pathways that evolved under natural selection. For example, non-natural formate converting pathways conceived and optimized *in vitro* have been shown to operate orthogonally to native metabolism^64^ and incorporate formate into biomass with higher yields than wildtype strains.^65^ Other examples are new-to-nature carbon fixation pathways that leverage atmospheric CO_2._^66^ Implementing such synthetic carbon fixation cycles in cell-free systems has enabled direct CO_2_ conversion to central metabolites, such as glycolate^67^ and malate,^68^ with rates rivaling naturally evolved processes like the Calvin cycle. These intermediates can also be upgraded to complex chemical products, such as the cell-free conversion of CO_2_ into a polyketide using a network of over 50 enzymes fine-tuned to minimize the loss of cofactors and intermediates.^12^ However, despite high efficiency and flexibility, the longevity of synthetic metabolic pathways currently remains limited *in vitro*.

Cell-free systems also simplify the implementation of bioinspired and non-natural energy regeneration.^69^ Although simple enzymatic production of ATP via dephosphorylation of phosphoenolpyruvate or creatine phosphate remains most common, new approaches run orthogonally to natural metabolism. This includes noncanonical cofactors, such as nicotinamide mononucleotide (NMN), which has provided reducing equivalents for purified and extract-based enzymatic reactions without disrupting or competing with natural NAD-dependent reactions.^70^ Alternatively, synthetic metabolism can mimic chloroplasts for light-powered ATP regeneration. Simple examples of chloroplast-like structures *in vitro* include vesicles with photosystem II and ATPase powering actin polymerization^71^ or vesicles with bacteriorhodopsin and ATPase powering cell-free protein synthesis.^72^ A more complex synthetic chloroplast combined thylakoid membranes with the CETCH cycle in microfluidic droplets for light-dependent regeneration of ATP and NADPH to power the cofactor-intensive CO_2_ fixation pathway.^73^ While solar energy is a powerful renewable resource harnessed by biological systems for millennia through photosynthesis,^74,75^ engineered approaches converting electrical energy into biological energy hold great potential to indirectly integrate any renewable energy source into biochemical processes.^76,77^ For example, electroenzymatic reduction of NADP^+^ mediated by a viologen-based hydrogel coupled to an electrode provided sufficient cofactor to enable CO_2_ reduction by crotonyl-CoA carboxylase/reductase.^78^ Electricity-powered ATP generation was also optimized with a synthetic enzymatic cascade to produce ∼400 μM ATP in 2 h, enough to drive transcription and translation *in vitro.*(79) The ability to use alternative energy sources and energy carriers in cell-free systems (e.g., noncanonical nucleotides, aromatic chemicals, nonnative ferredoxins) expands biosynthetic possibilities beyond cellular constraints into a vast opportunity space between biological and chemical methods. Transitioning these approaches from purified systems into cell extracts could also provide pivotal information for cellular implementation and/or lower cost *in vitro* systems as efficiency and scale continue to increase.^80^

## Machine Learning Will Increase the Yield and Robustness of Cell-Free Metabolism

Many of the improvements in cell-free gene expression and metabolism have come from stepwise optimization of reaction components and their concentrations to achieve higher yields.^4,30^ The increased prevalence of machine learning, design-of-experiments, and model-based predictions will accelerate the improvement of cell-free metabolism, particularly as *in vitro* experiments can rapidly generate the large sets of training data required for useful algorithms. Leveraging machine learning approaches will be essential for activating more diverse metabolic pathways and prolonging their activity *in vitro*, rather than relying on existing genome-scale models or fluxes derived from cells. Although cell extracts are able to mimic cellular functionality, there are inherent differences that limit direct comparisons between *in vitro* and *in vivo* metabolism.^3^ Differences include reduced regulatory layers (e.g., reduced genetic feedback loops and protein degradation), greater biochemical flexibility (e.g., tolerance of noncellular pH ranges and metabolite concentrations), increased toxicity thresholds, and the limited capability of cell-free systems to adapt, replicate, or maintain a state out of the thermodynamic equilibrium.^3^ This means cell-free metabolism is often shorter-lived than *in vivo,* with ATP generation sustaining protein synthesis for at most 24 h in batch reactions.^9^ However, some metabolic pathways in extracts and purified enzyme systems have demonstrated higher volumetric productivities than cells and reactions lasting multiple days.^6,81^ The longevity of biochemical reactions in cell-free systems may be increased by supplementing hypothesized limiting components (e.g., intermediates and cofactors)^69,82^ or by recapitulating the self-sustaining abilities of cells.^83,84^

Beyond these functional differences, cell-free systems are compositionally distinct from living cells because the contents of cell extracts are altered from a raw lysate (containing everything from a living cell) through centrifugation, incubation, and dialysis.^85^ The strategies of bottom-up construction and top-down derivation both result in complex parameter sets with interacting inputs and outputs that greatly benefit from computational approaches, which have significantly improved cell-free gene expression and can be extended to metabolism. For example, active learning approaches led to a 30-fold increase in gene expression in an *E. coli* cell extract,^86^ a 3-fold increase in expression from the minimal cell-derived extract,^20^ and rapid design of improved promoters for cyanobacteria.^87^ Similarly, Bayesian modeling accelerated the optimization of *B. megaterium* extracts^88^ and design-of-experiments improved *Pichia* cell-free expression up to 5-fold.^89^ Computational approaches also proved useful in optimizing bottom-up reconstituted cell-free systems. The composition of a synthetic cell-free carbon fixation cycle (CETCH, comprising 17 enzymes) was fine-tuned with multiple rounds of experiments requiring only a few days of experimental effort, resulting in 10-fold greater productivity and a 6-fold increase in efficiency.^5^ Similar machine learning and design-of-experiments approaches will be pivotal to improving newer cell-free systems that are less robust than PURE and *E. coli* extract. The diversity of approaches and systems summarized here merely scratches the surface of how machine learning can improve the performance of cell-free systems and move these systems beyond the natural limitations of cells. Future developments in the biochemical capabilities of cell-free systems and their outputs will benefit greatly from computational modeling and self-driving laboratories to test vast combinations of parameter sets and characterize *in vitro* biochemical networks more deeply.^90−92^

## Conclusion

Cell-free systems are powerful tools to study and engineer metabolism, both by mimicking natural pathways and by facilitating new opportunities for chemical transformations. Continued investment in cell-free synthetic biology will accelerate the study of unconventional microbes and the optimization of production strains, inform artificial cell design from the bottom-up and top-down, and enable new methods of combining metabolic features from disparate organisms. High-throughput, multivariate optimizations including machine learning approaches will be essential for achieving prolonged metabolic activity with more diverse pathways and reaction conditions. Together, these strategies will deepen our understanding of the living world and advance biological solutions for sustainable chemical conversion.
